# A Dual-Background Statistical Framework for Phosphoproteomics Highlights Intrinsic, High-Confidence Phosphorylation Signature by Mitigating Orthogonal Sources of Bias

**DOI:** 10.3390/proteomes14030033

**Published:** 2026-07-07

**Authors:** Bin Deng

**Affiliations:** Department of Biology, University of Vermont, Burlington, VT 05405, USA; bdeng@uvm.edu

**Keywords:** phosphoproteomics, R programming, DBE, motif logo, motif discovery

## Abstract

Background: Distinguishing genuine kinase–substrate motifs from background noise is a growing challenge, as mass spectrometry (MS)-based global phosphoproteomics identifies a rapidly expanding set of phosphorylation sites. One of the major limitations is selecting an appropriate background model that systematically controls both technical and biological sources of bias. Although using the entire proteome as a background in a FASTA format considers the overall amino acid composition, it is still prone to biases from protein abundance and the uneven distribution of sequence space (particularly around low-abundance proteins). By contrast, internal background methods can control experiment-specific detection biases, but they may not fully capture residue-specific compositions or general trends in phosphorylation. Methods: I develop a Dual-Background Enrichment (DBE) framework with a position-specific enrichment (PSE) strategy, which involves analyzing motif enrichment against two distinct background models: (1) A residue-heterogeneous internal background composed of phospho-motifs centered on the residue; e.g., phosphoserine (pS) motifs are tested relative to the pool of all detected phosphothreonine (pT) and phosphotyrosine (pY) motifs from the same experiment. (2) A FASTA background that includes all S, T, and Y residues in the UniProtKB proteome sequences. Results: Motifs are classified as high confidence if they meet statistical significance (q ≤ 0.05, fold enrichment > 1.5) against both background models. Conclusion: By applying the DBE strategy to a large-scale phosphoproteomics dataset, we distinguish motifs driven by amino acid composition (enriched in FASTA background only) from those reflecting kinase substrate specificity (enriched in both backgrounds). This dual-reference approach reduces false positives arising from sequence composition bias and enriches high-confidence candidate kinase recognition motifs.

## 1. Introduction

Phosphorylation is one of the most common post-translational modifications that plays a central role in cellular signaling. Large-scale phosphoproteomics provides the data necessary to understand these signaling networks, a process that depends on identifying overrepresented kinase-substrate sequence motifs and characterizing their quantitative changes across biological conditions. This approach involves analyzing short peptide sequences that are overrepresented around phosphorylation sites [1,2].

Although existing tools such as Motif-X 2.0/MoMo 5.5.9, PhosR (1.22.0), and PTM-SEA (2.0.0) support motif and kinase analyses, their results are highly dependent on the choice of background model. In practice, statistically establishing motif enrichment is typically performed using a single background (e.g., proteome-wide or user-defined), and systematic comparison between global and context-matched internal backgrounds is not commonly applied. With advances in phosphoproteomics, which enable the detection of over 81,000 unique sites of mouse tissues in a 12 h LC–MS/MS gradient [3], internal backgrounds are now sufficiently large to enable robust, context-specific motif enrichment analyses. Therefore, we implemented DBE to leverage both backgrounds’ advantages.

The accuracy of motif enrichment results relies heavily on the chosen background set, which must successfully model the noise floor. Two primary sources of bias can be met in this context:

- Global bias (FASTA background): Some amino acids are simply more common in the human proteome [4]. The complete proteome sequence may enrich motifs based on protein abundance or general sequence probability, rather than true kinase specificity [4].
- Experimental bias (internal background): While necessary, internal controls (e.g., non-phospho peptides) often fail to account for the major biases introduced by the phospho-enrichment and mass spectrometry (MS) detection steps, which similarly affect different residue types of S, T, and Y [5].

**Figure 1:**
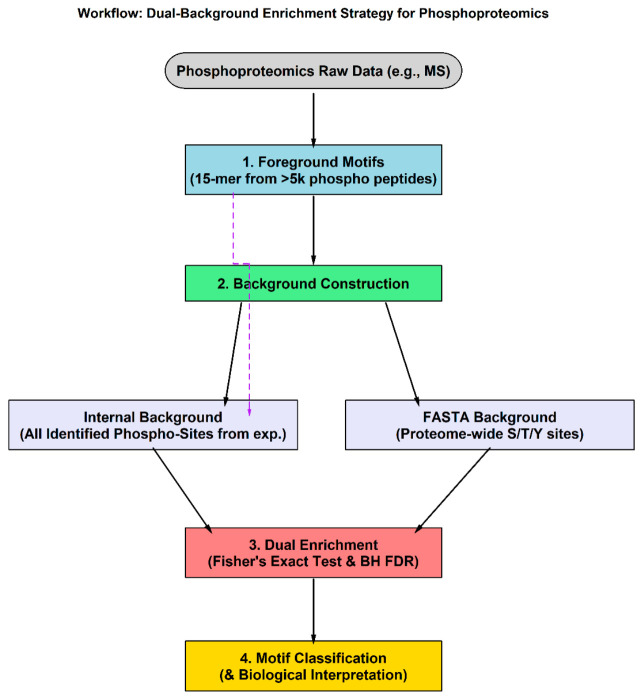
Dual-background enrichment (DBE) strategy for robust phosphorylation motif discovery.

Here, the DBE-PSE framework assesses enrichment of individual amino acids at specific positions surrounding the phosphorylation site. This approach enables the detection of statistically enriched residue–position associations while preserving local sequence context. Motif enrichment is subsequently evaluated using two distinct background models: (1) an internal positional background derived from experimentally observed phosphorylation sites and (2) a proteome-derived FASTA background constructed from the UniProt reference proteome. For a comprehensive analysis of phosphoproteomic motifs, a simplified R pipeline has been developed (Figure 1).

The pipeline includes peptide sequence cleaning, extraction of site-centered 15-mers, expansion of multi-site annotations, generation of DBE background enrichment, and comparison. Input files are accepted in the CSV file format without preprocessing, and the outputs consist of annotated motif tables and the results of enrichment comparisons through DBE frameworks.

## 2. Materials and Methods

### 2.1. Proteome Data Set

The file imaconly_multiconsensus_PeptideGroups (Phosphoproteome) was downloaded from the publication [6] and data access through Synapse (https://doi.org/10.7303/syn20820472, accessed on 1 May 2025). Briefly, all TMT-labeled samples had been enriched by IMAC for phosphorylated peptides and subsequently analyzed and quantified by LC-MS/MS using a Thermo Scientific Fusion Lumos mass spectrometer (Thermo Electron, San Jose, CA, USA). Data was processed using Proteome Discoverer 2.3 (Thermo Electron, San Jose, CA, USA). Detailed experimental information is included in the original publication. The Human Proteome Database (UniProtKB_proteome_UP000005640_AND_revi_2025_12_02, canonical proteins: 20,405) was downloaded from UniProt for use in FASTA background enrichment.

### 2.2. Phosphoproteomics Dataset Curation and Set Definition

All assigned phosphopeptides (*N*_Total_ > 70,000) were processed into 15-mer peptide sequences centered at the phosphorylation site (S, T, or Y). Two sets are defined for motif analysis based on this data:

- Foreground set (FG): The set of all assigned phosphomotifs corresponding to a specific residue type (i.e., all S motifs are the foreground when analyzing S motifs). The pipeline interrogates the primary sequence context surrounding these sites, meaning the discovered motifs reflect structural selection rather than differential abundance between conditions.
- Total assigned phospho-sites: The complete collection of all assigned phosphopeptides.

### 2.3. Background Set Construction

The DBE strategy requires two orthogonal background sets:

- Residue-heterogenous internal background (experimental control): When testing the foreground motifs for one residue (e.g., S), the internal background is defined as the set of motifs corresponding to all other assigned phosphopeptide residues (T + Y) in the experiment. Similarly, T FG tested vs. (S + Y); Y FG tested vs. (S + T). This set controls for technical biases common to phospho-enrichment and MS analysis across all detected phosphopeptides.
- FASTA background (proteome-wide control): Defined as all canonical S/T/Y sites extracted from the human proteome. This background controls for global sequence composition and amino acid frequency, enabling detection of motifs enriched beyond proteome-wide expectations.

### 2.4. DBE Strategy

The workflow is illustrated in Figure 1.

- Enrichment metric: For each potential motif *M*, a one-sided Fisher’s exact test [7] (pseudocount = 0.5) was used, and the analysis was performed independently against the two background models. Multiple testing correction was performed separately for each background type.

The contingency table was formulated as follows:

$$P=Fisher^{\prime }s\;exact\;test=\left(\begin{array}{cc} k_{fg\;} & n_{fg}-\;k_{fg}\\ k_{bg} & n_{bg}-\;k_{bg} \end{array}\right)$$

where $k_{fg\;}$ and $k_{bg}$ are the counts of the motif *M* in the foreground (experimental) and background (FASTA) datasets, respectively, and $n_{fg}$ and $n_{bg}$ are the total number of phosphosites in the respective datasets.

The resulting p-value represents the probability of observed motif distribution under the null hypothesis of random expectation.

For additional interpretability, the binomial probability of observed *K* or more motif occurrences under random conditions was computed as:

$$P(X\geq K)=\sum_{i=k}^{n}(\frac{n}{i})p^{i}{\left(1-p\right)}^{n-i}$$

where

*n*: total number of phosphopeptides in the foreground;

*K*: number of phosphopeptides containing the motif;

*p*: background probability of the motif (estimated from the background dataset);

*P*(*X ≥ K*): cumulative probability of observing at least K occurrences under a random distribution.

Internal test: Compares motif counts between the target residue and the internal background (e.g., S motifs vs. T + Y motifs).

FASTA test: Compares motif counts between the target residue and the global proteome background (e.g., S motifs vs. all S sites in the proteome).

- Statistical correction: The Benjamini–Hochberg (BH) procedure [7] was applied to the values resulting from both tests to determine the false discovery rate (FDR).

$$q_{\left(i\right)}=\frac{p_{\left(i\right)}\cdot m}{i}$$

where m is the total number of motif tests, and i is the rank of each p-value in ascending order.

- High-confidence filter: A motif was designated as high-confidence only if *q_Internal_* < 0.05 or *q_FASTA_* < 0.05 from either background. This combination of Fisher’s test with BH correction provides a reliable and interpretable assessment of motif significance while accounting for multiple hypothesis testing.

Enrichment fold was calculated by:

$$FE=\frac{\frac{a+p}{Nfg+2p}}{\frac{c+p}{Nbg+2p}}$$

where:

*a*: foreground motif count;

*c*: background motif count;

*p*: pseudocount.

Motifs were considered significant if they met either the high confidence (q < 0.05, fold enrichment > 1.5) or moderate confidence (q < 0.10, fold enrichment > 1.2) criteria.

- Motif logos: The DBE motif logos were constructed with the weighted matrix, which used a log-transformed enrichment weight (log2(FE)). This differs from a conventional position weight matrix (PWM) [8], which is derived from full sequence alignments. If a specific amino acid is defined at that position (e.g., a Proline P at position 9), the script calculates its log2(FE). It adds it to the running total for that specific amino acid row and position column. Once the matrix is compiled, motifs are then generated by the ggseqlogo R package (0.2.2). Because the weight at each position is the cumulative sum of log2(FE) values across all contributing significant motifs, the resulting logo height reflects both the enrichment magnitude of individual positional determinants and the number of significant motifs in which that amino acid–position association was detected.

## 3. Results

### 3.1. Extraction of Phospho-Motifs and Site-Level Annotation

The exported data “imaconly_multiconsensus_PeptideGroups” consists of a table containing 72,138 annotated peptide sequences that were directly exported from Proteome Discoverer software 2.3. To reduce the data size, I only keep the columns that include “annotated sequences”, “modifications”, “master protein accession numbers”, and “protein descriptions”, etc (Supplementary Table S1). The pipeline starts automatically to detect relevant columns and cleans peptide sequences by removing bracketed annotations (e.g., [Phospho], [+80], [Oxidation]) to recover the undecorated amino acid sequence. It then verifies that the cleaned sequences consist entirely of canonical amino acid characters, filtering out any entries that are empty or malformed after processing. Phosphorylation sites were then extracted from “annotated sequences” based on the information of “modification” strings and expanded so that each site was saved in an individual row. Each phospho-residue was annotated within the peptide sequence with a “pS/pT/pY” marking, and a 15-mer motif centered on the modified residue was generated. Assignment of unique site IDs using the gene name from the column “description” or “accession” if the gene name is missing. Peptides with ambiguous phosphorylation annotations (e.g., Phospho[S/T/Y]) were excluded from residue-specific foreground but were retained for background motif construction. For each ambiguous peptide, all plausible S-, T-, or Y-centered 15-mer motifs present within the peptide sequence were generated and included in the background, with sequence padding applied when candidate sites occurred near peptide termini. This ensured uniform representation of sequence context for downstream visualization and analysis.

**Figure 2:**
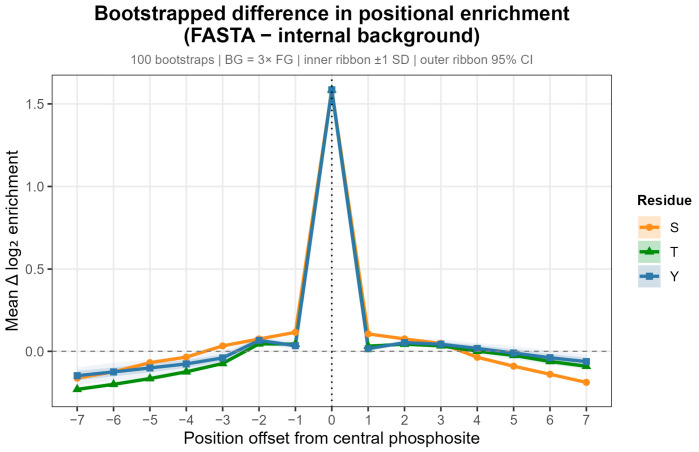
Central residue-specific enrichment by DBE frameworks. Bootstrapped difference in positional enrichment between FASTA and internal background definitions for S, T, and Y phosphosites. Lines represent the mean bootstrapped log2 enrichment difference (FASTA−internal) at each position relative to the phosphorylated residue (position 0); shaded bands indicate bootstrap variability. The dashed vertical line denotes the central phosphosite position.

A total of 38,953 unique 15-mer motifs were generated in the internal FG using R script A (v7.4_Internal_Background). This list consists of 33,218 motifs for S, 5190 for T, and 395 for Y, as detailed in Supplementary Table S2. An internal enrichment analysis has been conducted and saved for use in subsequent script B (v7.4_fasta_Background). FASTA background motifs were created by using the human proteome database. FASTA-based enrichment analysis was then performed, followed by a comparison and visualization of the internal and FASTA results. Figure 2 shows that all three residues (S/T/Y) exhibit a strong and sharply localized enrichment at the central position, with minimal background-dependent effects at the flanking positions. The sharp enrichment spike at position 0 is expected and confirms correct motif centering: because each foreground set is defined by its central residue type (e.g., all pS sites for the S analysis), the central position is perfectly discriminated against in the heterogeneous internal background. The biological signal of interest lies in the flanking positions (±1 to ±7), where the near-zero enrichment difference indicates that FASTA and internal backgrounds capture largely consistent positional sequence preferences across all three residue classes. This indicates that DBE is consistent, confirming the correct centering and specificity of the motif analysis.

### 3.2. DBE Refines Motif Enrichment Through Orthogonal Filtering

The application of the DBE framework significantly reduced the total number of assigned motifs by using enrichment criteria based on both FASTA and internal backgrounds. Comparative enrichment results are summarized in Table 1, and enrichment agreement metrics are presented in Supplementary Table S3.

**Figure 3:**
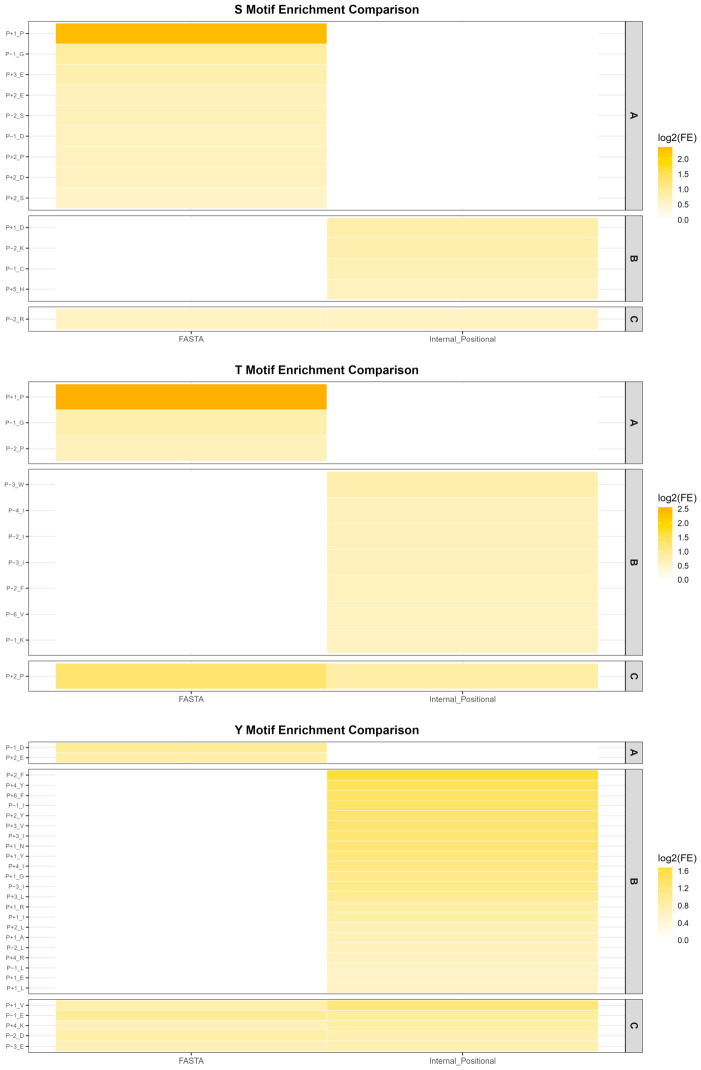
Comparative heatmaps of significant positional motifs enriched by the dual-background enrichment (DBE) framework for phosphoserine (S), phosphothreonine (T), and phosphotyrosine (Y) phosphorylation sites. Significant motifs were enriched using either a proteome-derived FASTA background or an internal positional background constructed from experimentally observed phosphorylation sites. Color intensity indicates log2FE. Motifs are grouped according to whether they were enriched exclusively by the FASTA background (A: FASTA Only), exclusively by the internal positional background (B: Internal Only), or by both background models (C: Shared, both significant). The overlap between background models decreases from pS to pY analyses, with pY motifs showing the greatest contribution from the internal positional background.

The heatmap (Figure 3) displays differential enrichments between the two backgrounds, which align with the results presented in Table 1. For pS motifs (top panel), nine motifs were enriched exclusively by FASTA enrichment, four were unique to the internal positional background, and one motif was shared between both approaches. FASTA-specific pS motifs were predominantly characterized by acidic and proline-associated residues surrounding the phosphorylation site, whereas internal-specific motifs displayed a more heterogeneous set of positional residue enrichments. For pT motifs (middle panel), overlap between the two enrichment strategies was limited to a single shared motif (P + 2_P). Three motifs were enriched exclusively by FASTA enrichment, whereas seven motifs were unique to the internal positional background. Several internal-specific pT motifs contained hydrophobic residues surrounding the phosphorylated threonine. The greatest divergence between enrichment strategies was observed for pY motifs (bottom panel). Twenty-two pY motifs were enriched exclusively by the internal positional background, compared with only two FASTA-specific motifs and five motifs shared between both approaches. Internal-specific pY motifs were enriched for hydrophobic and aromatic residues flanking the phosphotyrosine site. For detailed position-specific enrichment heatmaps, please see Supplementary Figure S1.

### 3.3. Motif Logo in the Comparison of DBE Significance

**Figure 4:**
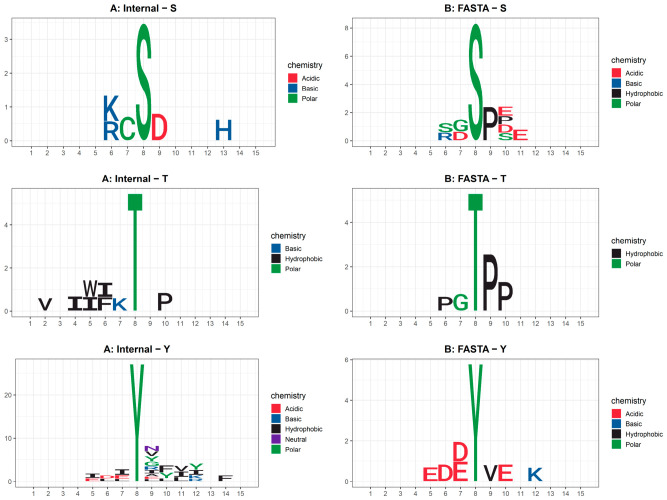
(**A**,**B**). Visualization of phosphosite motifs for serine (S), threonine (T), and tyrosine (Y) using different background models. Sequence logos represent position weight matrices (PWMs) of significantly enriched 15-mer flanking motifs centered on the modified phospho-acceptor residue (Position 8). Rows separate motifs by target residue: serine (S, top row), threonine (T, middle row), and tyrosine (Y, bottom row). Columns compare the two background baselines utilized by the DBE algorithm: sample-specific matching abundances (internal, left column A) and whole-proteome frequencies (FASTA, right column B). The X-axis represents the relative amino acid positions flanking the central phosphosite (P1 to P15). The Y-axis denotes cumulative enrichment strength derived from log2 FE scores. Amino acids are color-coded by chemical properties: acidic (red), basic (blue), hydrophobic (black), and polar (green).

In Figure 4, the motif logos of pS, pT, and pY represent the statistical significance of amino acid enrichment (y-axis) at each position against a FASTA, internal, or both backgrounds. For pS motifs, the enrichment of acidic residues flanking pS sites is consistent with substrate preferences reported for acidophilic kinases such as Casein Kinase 2 (CK2) [9]. For pT motifs, the high number of proline-containing sequences suggests activity from proline-directed kinases like MAPKs [10] and CDKs [2], even though the observed motif architecture does not uniquely support any individual kinase family. For pY motifs, which are rare and make up only about 1% of the phosphoproteome (compared to 99% for serine/threonine), the internal positional background showed a high amount of hydrophobic and aromatic residues around the phosphorylation sites, commonly associated with selective tyrosine kinase substrate recognition. Although motif enrichment alone cannot assign individual kinases, these observations suggest that the internal positional background improves the detection of biologically relevant kinase-recognition signatures that are partially obscured by conventional proteome-wide background models.

### 3.4. The Motifs Enrichment Comparison Between DBE and the MotifX

To compare motif discovery performance, we tested both DBE and MotifX on the same phosphosite foreground (Fg) dataset. For MotifX, we used an Fg threshold of 10 and kept all other default settings. We only included motifs with enrichment scores above 20 in the comparison (Supplementary Table S4).

From Table 2, there are 39 pS motifs and 10 pT motifs in MotifX only. In comparison, DBE exclusively found three pS motifs and seven pT motifs. Although both tools enriched a similar number of pT motifs, only four motifs were shared between them. The biggest difference was with pY motifs. DBE found 29 enriched pY motifs, but MotifX found zero under these settings. Notably, even after lowering the MotifX enrichment score cut-off from 20 to 0 (default), the method detects only a single pY motif.

While the two methods yielded 15 shared motifs out of the 54 total DBE features, providing a 27.8% orthogonal validation rate, these overlapping features ultimately reveal two highly robust kinase substrate signatures confirmed by both computational frameworks. For pS, proline at +1 is the strongest shared signal that defines proline-directed kinases like CDKs and MAPKs, and acidic (D/E) at −1, +1, +2, +3 defines the CK2 acidophilic recognition sequence. For shared pT, P at −2, +1, +2 is exclusively proline-directed for CDK1 and MAPKs.

## 4. Discussion

### 4.1. DBE Comparison to MotifX

Both the iterative MotifX algorithm [8] and the custom DBE framework were applied to the same set of experimentally assigned phosphorylation sites. The limited overlap between DBE and Motif-X motifs suggests that the two methods interrogate phosphorylation sequence patterns using fundamentally different statistical frameworks.

First, MotifX relies on a single global background (FASTA) to evaluate foreground S, T, and Y sites against all corresponding S, T, and Y sites across the reference proteome. In contrast, DBE utilizes dual backgrounds to cross-evaluate each modified residue class against a background composed of the alternative classes (i.e., enriching S against a T + Y background, T against S + Y, and Y against S + T).

Second, MotifX employs a binomial probability model along with a conservative *p*-value cutoff (typically 10^−6^) and a minimum occurrence threshold to limit false discoveries. In contrast, the DBE framework uses Fisher’s exact test with the Benjamini–Hochberg (BH) correction to control the false discovery rate (FDR), reporting q-values for each motif comparison.

Third, both MotifX and DBE implement a position-specific strategy, but MotifX relies on an iterative and recursive extraction algorithm. This approach actively searches for co-occurring combinatorial patterns, which lock multiple fixed positions into a single consensus string. In contrast, DBE evaluates independent positional features across the entire dataset without nesting constraints. Therefore, to enable a direct comparison, MotifX motifs must be parsed into the single-position format used by DBE, resulting in 253 motif-feature relationships (Supplementary Table S5) in the complete comparison table. A final comparison between DBE and MotifX revealed 103 enriched unique residue-position features (Table 2).

MotifX enriches overrepresented consensus motifs relative to a proteome-derived background and is particularly effective at detecting dominant phosphorylation signatures. Because pS sites constitute the majority of phosphoproteomic datasets, motif discovery is often driven by pS-associated sequence patterns. In contrast, DBE incorporates residue-class-specific positional enrichment, in which pS, pT, and pY sites are evaluated relative to one another. As a result, DBE shifts the analytical focus from determining whether a motif is enriched relative to the proteome to identifying sequence features that distinguish one phosphorylation class from another. This targeted approach directly accounts for the discovery of 29 enriched pY motifs that were otherwise masked by MotifX.

### 4.2. Rationale for Dual-Background Correction

The DBE framework was developed to address two distinct sources of bias that influence phosphorylation motif discovery. The key strength of our approach is the combination of these filters:

(1) Internal positional background (experimental and context-specific bias correction): DBE used an internal background correction/cross-site normalization to improve specificity, where phosphorylation on one residue type is analyzed against another to detect true sequence preferences, similar in principle to comparative frameworks used in ProteoViz [11], GSEA analyses [12], and PTM-SEA [13]. By this method, many technical biases (ionization, enrichment efficiency, instrument sensitivity, and peptide detectability) are controlled because foreground and background come from the same experiment (sample preparation, enrichment, and LC-MS/MS). Basically, phosphorylation events centered on one residue type are evaluated relative to phosphorylation events centered on the remaining residue classes. Consequently, motifs enriched through internal positional enrichment represent sequence features that distinguish one phosphorylation class from other experimentally observed phosphorylation events rather than from the proteome as a whole. (Supplementary Table S3).
(2) FASTA background (proteome-wide composition correction): The UniProtKB proteome (UP000005640) served as a reference background to correct for global amino acid frequencies. This approach minimizes baseline biases due to natural residue abundance, ensuring that the enriched motifs reflect true biological selection rather than background proteome composition. Such proteome-wide models are standard in motif enrichment pipelines to isolate rare sequence patterns preferentially targeted by specific enzymatic or regulatory pathways.
(3) Complementary information revealed by dual background analysis: Comparison of FASTA and internal positional enrichment results demonstrated that the two background models capture overlapping but distinct motif populations. Shared motifs likely represent robust phosphorylation signatures that are independent of background selection, whereas FASTA-only and internal-only motifs highlight sequence features that are preferentially detected under different reference frameworks. Notably, differences between enrichment strategies increased progressively from pS to pT and were most pronounced for pY motifs. Phosphoserine motifs showed contributions from both FASTA and internal positional enrichment, highlighting that many pS sequence preferences are detectable relative to both proteome-wide and experimentally derived backgrounds. In contrast, pT motifs exhibited greater enrichment within the internal positional framework, implying increased sensitivity for detecting context-dependent phosphorylation signatures. The pY motifs stood out the most. Their strongest enriched signatures appeared exclusively when using the internal positional control. These motifs frequently featured hydrophobic and aromatic residues flanking the phosphotyrosine site, revealing an architectural preference that global proteome comparisons easily obscure.

Overall, these findings prove that the FASTA and internal positional frameworks are complementary rather than redundant. FASTA modeling enriches overrepresentation relative to the global proteome composition, whereas internal positional analysis isolates localized sequence preferences unique to the observed experiment samples. The integration of both approaches through the DBE framework expands motif discovery and provides a more comprehensive characterization of phosphorylation site sequence determinants than either background model alone.

### 4.3. DBE Framework’s Limitations and Future Work

Although the DBE framework establishes a strict baseline for obtaining enriched phosphorylation features, its application has notable limitations. First, the DBE framework requires a minimum dataset depth to ensure adequate statistical power for both the FASTA and internal positional background comparisons. The internal positional background is particularly sensitive to dataset size because it relies on the pool of complementary phosphoresidue classes (e.g., pT and pY sites serve as the internal background when testing pS motifs). If any residue class is too sparsely represented, the background pool becomes insufficient to support robust Fisher’s exact test estimation at individual positions. Second, motif extraction is highly sensitive to the selected reference background. DBE addresses this dependency by executing parallel comparisons against both proteome-derived FASTA background and experiment-derived internal positional background. Because these dual models evaluate entirely different aspects of enrichment, motifs captured exclusively by a single background demand context-specific interpretation. These operational discrepancies further emphasize that computational predictions must ultimately be paired with experimental validation to verify actual biological relevance. Third, DBE is designed to find statistically enriched sequence features surrounding phosphorylation sites and does not directly assign upstream kinases or signaling pathways. Consequently, our rule-based kinase predictions provide preliminary hypotheses but require integration with complementary tools such as kinase-substrate databases (e.g., PhosphoSitePlus [14]), kinase enrichment analysis (KEA) [15], or experimental validation via kinase inhibitor assays. The future work will focus on biological validations, as well as empirical validation using synthetic phosphopeptides with well-characterized kinase recognition motifs added to standard cell lysates (e.g., HeLa or *E. coli*), followed by LC-MS/MS phosphoproteomics and comparison of DBE motif recovery against ground-truth sequences. Finally, the current implementation is restricted to the primary sequence flanking each phosphosite. Future updates will move beyond local sequence by incorporating protein structures, evolutionary conservation, and interaction networks to provide a more holistic view of discovered motifs.

Importantly, motif enrichment at the sequence level does not account for the regulation of specific proteoforms. Distinct biological roles are frequently driven by specific splice variants, shifting phosphorylation states, or combinations of different post-translational modifications (PTMs). Conventional motif analysis often overlooks these variables by isolating individual modification sites rather than viewing them within the context of the whole protein.

## 5. Conclusions

In this study, we developed a DBE-PSE framework for phosphorylation motif analysis. By integrating a proteome-derived FASTA background with an experimentally derived internal positional background, DBE provides complementary perspectives on phosphorylation site sequence architecture and improves the enrichment of phosphorylation-specific sequence determinants. Furthermore, DBE should be viewed as a complementary positional motif enrichment framework that extends conventional consensus motif discovery approaches rather than replacing established methods such as MotifX. Whereas MotifX excels at generating concise consensus motifs, particularly for pS, which summarize dominant phosphorylation patterns, DBE provides a statistically rigorous framework for enriching and quantifying individual positional determinants and residue-specific sequence preferences, particularly for pY motifs. Together, these approaches offer complementary perspectives on kinase–substrate recognition and phosphorylation site context.

## Figures and Tables

**Table 1:** Summarizes the comparative enrichment between FASTA and internal backgrounds.

Residue	FASTA Only	Internal Only	Both Significant
pS	9	4	1
pT	3	7	1
pY	2	22	5

**Table 2:** Comprehensive STY motif distribution and background analysis between DBE and MotifX.

Category	pS	pT	pY	Total
Shared (DBE + MotifX)	11	4	0	15
DBE only	3	7	29	39
MotifX only	39	10	0	49

## Data Availability

The data training and demonstrations of the R program (R 4.5.0) are applied in R Studio RStudio/2026.05.0+218 Chrome. Example datasets and all R scripts required to reproduce the DBE pipeline are available on Zenodo (https://doi.org/10.5281/zenodo.17545060, accessed on 25 June 2026). The raw mass spectrometry data are publicly available under the PRIDE accession number PXD015690.

## Supplementary Materials

The following supporting information can be downloaded at https://www.mdpi.com/article/10.3390/proteomes14030033/s1. Figure S1: Position-specific enrichment heatmaps generated using Internal Positional and FASTA background models; Table S1: Imaconly_multiconsensus_PeptideGroups_modified; Table S2: FG_Sites_Explicit; Table S3: Enrichment_Significant_Motifs; Table S4: MotifX STY scores above 20; Table S5: Comparison_Side_by_Side (DBE vs. MotifX).
