# Supplementary material for: A Dual-Background Statistical Framework for Phosphoproteomics Highlights Intrinsic, High-Confidence Phosphorylation Signature by Mitigating Orthogonal Sources of Bias
Source: Proteomes. 2026 Jul 7;14(3):33. doi: 10.3390/proteomes14030033 (PMC13398324; doi:10.3390/proteomes14030033)
Supplement: Supplementary file 1 [file proteomes-14-00033-s001.zip › Supplementary Figure S1_Position-specific enrichment heatmaps.pdf]

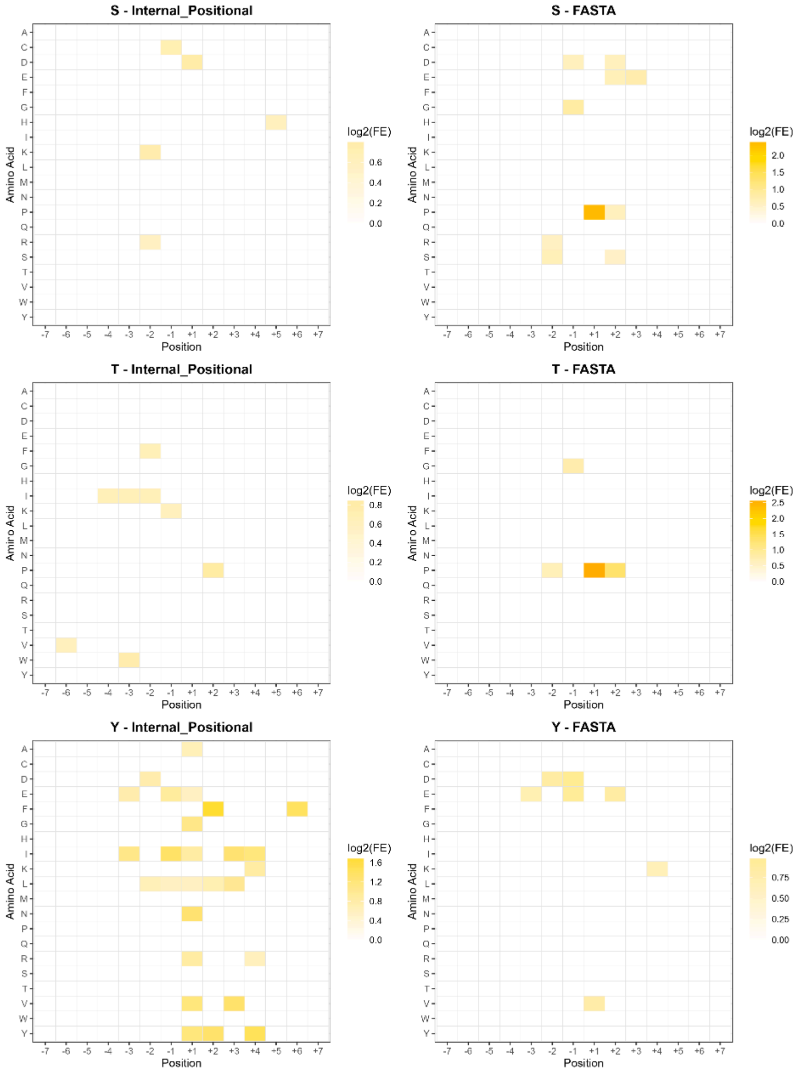

Supplementary Figure S1. Position-specific enrichment heatmaps generated using Internal Positional and FASTA background models.

Heatmaps display significantly enriched amino acid residues at positions surrounding phosphoserine (pS), phosphothreonine (pT), and phosphotyrosine (pY) sites. Rows represent amino acids and columns represent positions relative to the central phosphorylation site (−7 to +7). Color intensity indicates log2 fold enrichment (log2FE), with darker colors representing stronger enrichment. The left panels show enrichment relative to the Internal Positional background, whereas the right panels show enrichment relative to the proteome-derived FASTA background. Comparison of the two background models reveals both shared and background-specific positional sequence preferences surrounding phosphorylation sites.

The heatmaps displayed distinct enrichment patterns among pS, pT, and pY phosphorylation sites and highlighted differences between the Internal Positional and FASTA background models. For pS sites, both enrichment strategies identified several positional preferences, although the FASTA background detected stronger enrichment signals overall. For pT sites, the Internal Positional background identified

additional enriched residues that were less apparent in the FASTA analysis. The greatest divergence was observed for pY sites, where Internal Positional enrichment revealed substantially more positional determinants than the FASTA background.
